# Target Prediction of 5,10,15,20-Tetrakis(4′-Sulfonatophenyl)-Porphyrin Using Molecular Docking

**DOI:** 10.3390/pharmaceutics14112390

**Published:** 2022-11-05

**Authors:** Ana-Maria Udrea, Andra Dinache, Angela Staicu, Speranta Avram

**Affiliations:** 1 Laser Department, National Institute for Laser, Plasma and Radiation Physics, Atomistilor 409, 077125 Magurele, Romania; 2 Research Institute of the University of Bucharest—ICUB, University of Bucharest, 91-95 Splaiul Independentei, 050095 Bucharest, Romania; 3 Department of Anatomy, Animal Physiology and Biophysics, Faculty of Biology, University of Bucharest, 91-95 Splaiul Independentei, 050095 Bucharest, Romania

**Keywords:** molecular docking, in silico, cancer therapy, cancer protein target, BCL-2 family protein, PDT, HSA, binding affinity, UV–vis absorption spectroscopy

## Abstract

Photodynamic therapy has the potential to be a new and effective cancer treatment. Even if in vitro and in vivo research show promise, the molecular mechanism remains unclear. In this study, molecular docking simulations predict the binding affinity of the 5,10,15,20-tetrakis(4′-sulfonatophenyl)-porphyrin tetraammonium photosensitizer on several potential targets in photodynamic treatment. Our results indicate that this photosensitizer binds to several receptor targets, including B-cell lymphoma 2 (BCL-2) and other related proteins BCL-xL, MCL-1, or A1. The binding affinity of the porphyrin derivative with human serum albumin was determined using UV–vis absorption spectroscopy and predicted using molecular docking. We conclude that the studied porphyrin photosensitizer binds to human serum albumin and may inhibit the cancer cell line through its interactions with HIS and MET AA residues from BCL-2, MCL-1, and β-catenin receptors or through its low estimated free energy of binding when interacting with A1 and BCL-B receptors.

## 1. Introduction

Cancer is the leading cause of death around the globe. In 2020, 324,635 new cases of melanoma were recorded, and 57,043 fatalities were due to the disease [1]. Melanoma is a type of skin cancer with high multidrug resistance, risk of mortality, and recurrence [2]. Surgery, radiation, chemotherapy, and biological therapy are the standard treatments for melanoma [2]. 

Photodynamic therapy (PDT) is a potential cancer treatment option that is effective even for melanoma patients. This process requires the use of a visible light source to photoactivate a sensitizer. The sensitizer must be safe, and its toxicity must be achieved only when it is photo-activated due to generation of reactive oxygen species (ROS) such as singlet oxygen. Photodamage to proteins can occur in the photosensitizer (PS) region due to high levels of ROS resulting in tumour cell death [3,4,5]. 

Porphyrin-based photosensitizers are among the most widely studied compounds used in PDT [6,7,8].

In a prior study, we concluded that 5,10,15,20-tetrakis(4’-sulfonatophenyl)-porphyrin tetraammonium (TPPS) is an effective photosensitiser [9]. We explored the mechanisms that cause apoptosis in the Mel-Juso melanoma cell line after incubation with TPPS-functionalized iron oxide nanoparticles (γ-Fe2O3 NPs TPPS) followed by irradiation, and we came to the following conclusions:

First, caspase-3 is active, and pro-caspase-3 levels are reduced, implying that apoptosis is being initiated. However, with the low increased levels of Bcl-2-associated X protein (Bax), caspase-3 activation is most likely achieved through multiple pathways.

Second, the decrease in mini-chromosome maintenance complex component 2 (MCM-2) protein levels may lead to melanoma cell death, decreased tumour development, and cell adhesion.

Third, the decrease in β-catenin protein levels may contribute to apoptosis induction, proliferation suppression, and cell adhesion reduction [9].

Generation of ROS for γ-Fe2O3 NPs TPPS is higher than in the case of TPPS alone due to the capacity of NPs to transport the photosensitizer intracellular [9]. 

Cell death occurs via apoptosis when PS is found in the mitochondria, necrosis when PS is found in the cell membrane, and autophagy when PS is found in the lysosomes [10]. When the PS is found in mitochondria, lysosomes, or the endoplasmic reticulum, it causes apoptosis by oxidative stress [11].

Still, the apoptotic molecular targets in PDT are yet unclear; however, some studies identify possible pathways [6,12]. The short biological half-life and narrow area of impact in live cells yield singlet oxygen, indicating its intense reactivity toward proteins, and other biochemical substrates [13]. Because singlet oxygen has a short migration time, we know that the sensitizers should be close to the target [14].

Given the high-rate constants for reactions with ROS, several proteins are viewed as one of the main PDT targets [15]. BCL-2 family proteins permeabilize the outer membrane of the mitochondria to regulate cell death [16,17]. Tumour necrosis factor receptor superfamily member 6 (Fas) and Nuclear factor kappa-light-chain-enhancer of activated B cells (NFKB) are proteins identified as possible targets in PDT that are linked to apoptosis [18]. 

The Eukaryotic Translation Initiation Factor 2 Alpha Kinase 1 (EIF2AK1) enzyme mediates the phosphorylation of eukaryotic Translation Initiation Factor 2A (eIF2α), which causes stress to the endoplasmic reticulum and leads to apoptosis upstream of the mitochondrial pathway [19]. Abnormal levels of β-catenin are linked to various malignancies, including melanoma. β-catenin inhibition may slow tumour growth [9,20]. Fas is a cell surface death receptor involved in PDT; some studies indicate that this receptor is overexpressed after PDT [11,21].

This study aims to elucidate the mechanism of action, the pharmacogenomics, and pharmacokinetics of TPPS as a photosensitizer that may be used in melanoma cancer treatment.

Since the specific mechanism of action is yet unknown, using our in silico expertise [22,23,24], we predicted the pharmacodynamic profile of the TPPS. Consequently, using molecular docking, we calculated the interaction of TPPS with the BCL-2 protein family, namely the β-catenin, NFKB, Fas, and EIF2AK1 proteins.

Kessel and Castelli’s in vitro study identified BCL-2 as the target of 9-capronyloxy-tetrakis (methyoxyethyl) porphycene (CPO) that induces a rapid apoptotic response [17].Therefore, we compare the TPPS activity on the BCL-2 receptor with the one of CPO.

Moreover, we used Swiss Similarity, a web service that conducts a ligand-based virtual screening of multiple libraries of small compounds to identify an approved clinical drug with the highest similarity with TPPS [25]. The interaction of the drug identified with Swiss Similarity and the protein targeted in this study was also computed and compared with the molecular docking results of TPPS.

The predominant plasma protein in humans is serum albumin (HSA). HSA is a single-chain protein with three homologous domains separated into two subdomains (A and B). HSA is an important drug transporter in plasma that may bind both endogenous and exogenous compounds [26]. The interaction of TPPS with HSA is critical for pharmacokinetics and in vivo effectiveness. Consequently, we also predicted the interactions between TPPS and HSA using molecular docking simulations. Furthermore, using UV–vis absorption spectroscopy, we determined the binding affinity of TPPS when it interacts with HSA.

Moreover, the pharmacogenomics and absorption, distribution, metabolism, excretion, and toxicity (ADMET) of TPPS is calculated with ADMETlab 2.0 web service [27]. SuperCYPsPred web tool was also used to predict the cytochrome activity of TPPS [28]. Other possible targets of TPPS were predicted using the SuperPred web service [29]. 

To summarise, this study intended to predict the pharmacodynamics of TPPS when interacting with cancer therapy protein targets and to evaluate its interaction with HSA using molecular docking and UV–vis absorption spectroscopy. The TPPS pharmacogenomics and pharmacokinetics were also predicted and compared with the computed values of CPO and clinically used PS temoporfin. 

## 2. Materials and Methods

### 2.1. Materials

TPPS was purchased from PorphyChem SAS, Dijon, France, and its purity was >95%. HSA (human serum albumin) was purchased from Sigma Aldrich (St. Louis, MI, USA), purity > 96%. Stock solutions of the two compounds were prepared in ultrapure water at 4 × 10^−6^ M for TPPS and 3 × 10^−6^ M for HSA. Seven samples were prepared containing a fixed concentration of TPPS (1.5 × 10^−6^ M) and varying the HSA concentrations from 0 to 2 × 10^−6^ M. After preparation, the samples were left for 2.5 h at room temperature (24 °C), in dark, before analysis. 

### 2.2. Molecular Target Selection and Similarity Virtual Screening

To predict the mechanism of action of TPPS, we selected proteins from the BCL-2 family and other proteins involved in PDT or cancer therapy (Table 1).

We have used the 3D structures of proteins from the RCSB Protein Data Bank (PDB) [30] (Table 1) and prepared them for molecular docking analysis. Where the experimentally obtained protein structures were not available, we used Alpha-Fold to predict the structures [31,32]. 

All small molecules were 3D-protonated and energy-minimised using forcefield MMFF94X at a 0.01 gradient and Gasteiger (PEOE) partial charges from MOE software [33,34,35,36].

Since the four cations (NH_4_^+^) of the 5,10,15,20-tetrakis(4’-sulfonatophenyl)-porphyrin tetraammonium considerably cumber our molecular docking model, we did not consider them in simulations, using instead the anionic compound. The role of NH_4_^+^ in the porphyrin structure is to improve solubility and not influence the binding interaction. 

The Swiss Similarity ligand-based virtual screening was used to predict the clinically used compound with the highest TPPS similarity.

For the Autodock simulations, we converted the small molecules into the .pdbqt format using Open Babel [37].

### 2.3. Pharmacodynamic, Pharmacogenomic, and Pharmacokinetic Predictions

For the pharmacokinetic predictions, we used ADMETlab 2.0, a web-based application for calculating ADMET properties in an accurate and thorough manner [27]. 

We used the SuperCYPsPred web tool to predict cytochrome activity. This web server uses both MACCS and Morgan fingerprints [28]. 

To predict the possible targets of TPPS, we used the SuperPred web service that correlates chemical similarities between molecules with molecular targets and treatments [29]. The results were compared with the predictions made for temoporfin. 

For the predictions, we used the SMILES format of TPPS, CPO, and temoporfin compounds.

### 2.4. Molecular Docking

We used Autodock 4.2.6 software for the docking studies [38]. For the predictions, the grid box was selected to contain the whole protein to perform blind docking; the grid points spacing, the grid point dimension, and the Cartesian coordinates of the central grid point of the map are different for each protein (Table 1). 

We used genetic algorithm (GA) search parameters (with which we generated 100 confirmations for each protein–ligand interaction), and the file was saved as Lamarckian [39]. 

**Table 1:** Selected targets in PDT used in molecular docking approach, protein databank code (PDB), the grid points spacing (GPS), chain used, grid points in dimension (GPD), and coordinates of central grid point of the map (GPM) for each model.

Target	Identification Code	GPS	Blind Molecular Docking	
			GPD (x y z)	GPM (x y z)
BCL-2	PDB 2XA0 [40]	0.375 Å	112; 106; 96	33.111, −12.383, −15.527
BCL-B	PDB 4B4S [41]	0.375 Å	112; 126; 116	−11.425, 20.511, 7.450
BCL-xL	PDB 3WIZ [42]	0.519 Å	126; 100; 126	40.398, 2.687, −22.529
MCL-1	PDB 3WIX [42]	0.375 Å	108; 122; 98	−9.969, 2.268, −48.521
A1	PDB 5UUL [43]	0.375 Å	116; 124; 102	−9.348, 5.397, −5.604
BCL-W	PDB 2Y6W [44]	0.419 Å	100; 124; 92	−22.718, 9.756, −4.104
β-catenin	PDB 1LUJ [45]	0.853 Å	78; 70; 126	23.902, 31.829, 33.523
NFKB	PDB 1SVC [46]	0.536 Å	126; 126; 126	40.385, 8.741, 38.710
Fas	PDB 1DDF [47]	0.375 Å	126; 94; 84	1.736, −1.315, 2.392
EIF2AK1	AF-Q9BQI3-F1 [31,32]	0.525 Å	126; 126; 126	11.643, −1.149, −3.074
HSA (blind)	PDB 1N5U [48]	0.658 Å	126; 78; 126	24.950, 5.672, 19.674

### 2.5. UV–Vis Absorption Spectroscopy

UV–vis absorption spectra of samples were recorded with a Lambda 950 UV–Vis-NIR spectrophotometer (PerkinElmer, Inc., Waltham, MA, USA), between 200 and 600 nm, in 10 mm quartz cuvettes. 

## 3. Results

### 3.1. Small Molecules and Similarity Report

Swiss Similarity virtual screening revealed that temoporfin (Table 2) has the highest similarity to TPPS and is already approved for head and neck squamous cell carcinoma treatment [49]. Temoporfin has a similarity score of 0.668 with TPPS. Therefore, we also predicted the interactions with our selected protein targets. 

The 2D structures of TPPS, temoporfin, and CPO and the SMILES codes of those compounds are presented in Table 2. 

### 3.2. Pharmacodynamic, Pharmacogenomic, and Pharmacokinetic Predictions

We predicted the interaction of the cytochromes CYP1A2, CYP2C19, CYP2C9, CYP2D6, and CYP3A4 with TPPS, temoporfin, and CPO using the SuperCYPsPred web service [28] (Table 3).

As shown in Table 3, according to MACCS fingerprint predictions, TPPS and temoporfin are inactive on CYP1A2, CYP2C19, CYP2D6, and CYP3A4 and active on CYP2C9 according to MACCS fingerprint but inactive on all CYPs according to Morgan fingerprint (Table 3). According to both fingerprints, CPO is inactive on CYP1A2, CYP2C19, CYP2D6, CYP3A4, and CYP2C9.

The target predictions computed with SuperPred [29] show that both temoporfin and TPPS may interact with several targets. In this study, we present only the therapeutic targets relevant to melanoma cancer therapy (Table 4). The temoporfin target with the highest probability (96.37%) is the DNA (apurinic or apyrimidinic site) lyase; as shown in Table 4, glioma, melanoma, ocular cancer, and solid tumours are the therapeutic indications (Table 4). The TPPS target with the highest probability (95%) is the thymidylate synthase as shown in Table 4, and gastric adenocarcinoma is the therapeutic indication (Table 4).

ADMET predictions were also made for TPPS compared with temoporfin and CPO porphyrin derivatives using ADMETlab 2.0 and are presented in Table 5 [27].

According to the predictions, none of the medications had a high possibility of inhibiting hERG and binding to the androgen receptor ligand-binding domain or peroxisome proliferator-activated receptor gamma (Table 5). TPPS, CPO, and temoporfin produce mitochondrial membrane potential, bind P53 and the oestrogen receptor ligand-binding domain, and cause respiratory toxicity. TPPS but not CPO or temoporfin induces hepatotoxicity in humans. 

CPO and temoporfin have a high probability of producing drug-induced liver injury and binding to the aryl hydrocarbon receptor, whereas TPPS’s probability is low. TPPS and temoporfin have a low carcinogenicity risk, whereas CPO has a high risk. For TPPS and CPO, the heat-shock factor response element is low, while for temoporfin, it is high. The results also show that porphyrin derivatives do not violate the acute-toxicity criteria (Table 5).

### 3.3. Molecular Docking

The lower the estimated free energy of binding (EFEB) (kcal/mol) or the estimated inhibition constant (*K_I_*), the more likely the ligand will bind to that target [50]. We used Autodock 4.2.6 software to predict the EFEB and the inhibitory constant (*K_I_*) of TPPS when interacting with biological target proteins BCL-2, BCL-B, BCL-xL, MCL-1, A1, BCL-W, β-catenin, NFKB, Fas, and EIF2AK1. Except for EIF2AK1, where we used the Alpha Fold predicted model, we used the crystal structures imported from PDB (Table 6). 

**Figure 1:**
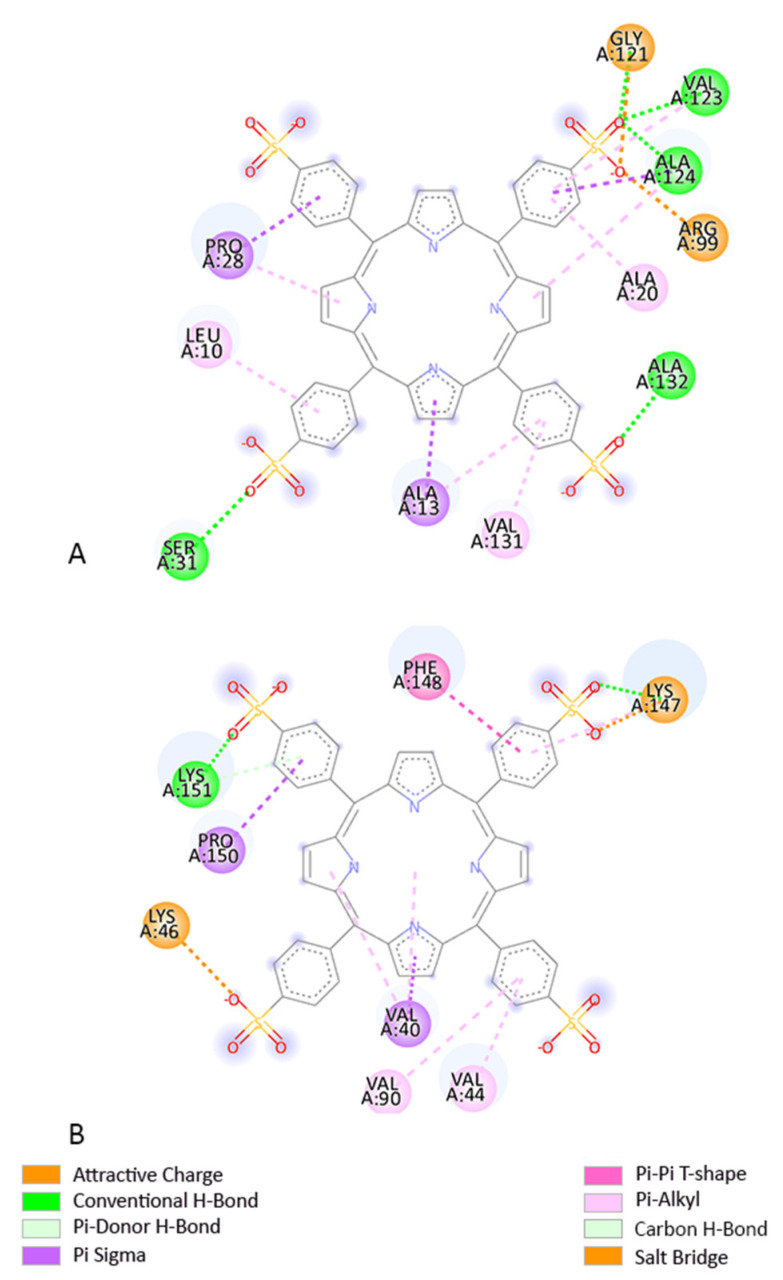
Two-dimensional structures of TPPS and amino acid (AA) residues from its binding site when interacting with (**A**) BCL-B receptor and (**B**) A1 receptor.

Our predictions show that TPPS had the lowest EFEB when interacting with A1 (−14.99 kcal/mol) and BCL-B (−11.49 kcal/mol), according to the Autodock prediction (Table 6), (Figure 1). However, in general, TPPS has a good predicted interaction with the proteins studied given by the values of EFEB lower than −6 kcal/mol [39] as shown in Table 6. 

The Autodock simulation shows that the CPO photosensitizer had the lowest EFEB of −10.22 kcal/mol when interacting with the BCL-2 receptor. This value is higher than the predicted value of TPPS (Table 6). Yet, if we compare the results obtained using the SwissDock webserver, CPO has the lowest EFEB of −8.94 kcal/mol, and TPPS has the lowest EFEB of −10.41 kcal/mol (Table 6).

### 3.4. Determination of the Binding Affinity through UV–Vis Absorption Spectroscopy

**Figure 2:**
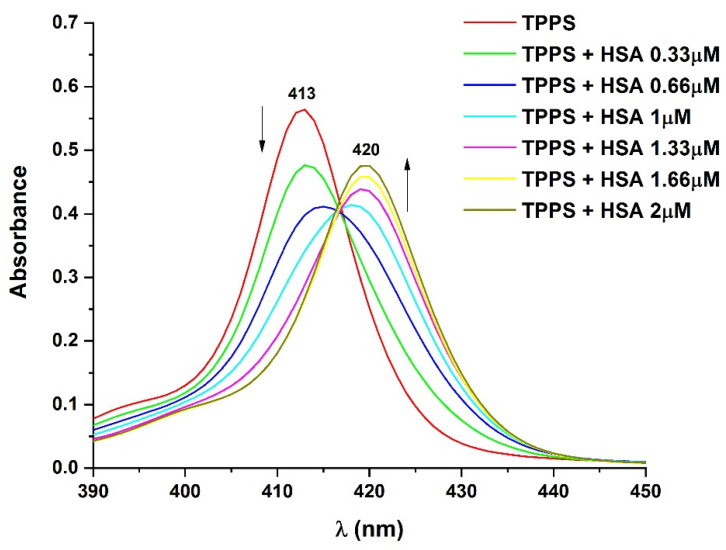
UV–vis absorption spectra of TPPS at a concentration of 1.5 µM and HSA with concentrations varied between 0 and 2 µM.

The recorded UV–vis absorption spectra of the samples (Figure 2) show how the spectral properties change with increasing the concentration of HSA (HSA), while the concentration of TPPS (TPPS) was kept unmodified. 

The UV–vis absorption spectrum of TPPS presents an absorption band with maximum at 413 nm (the Soret band) and the Q bands, at higher wavelengths, with maxima at 518 nm, 555 nm, and 583 nm. The positioning of the Soret band of TPPS at 413 nm indicates that, at this concentration, the solution contains only TPPS monomers. 

HSA has one absorption band with maximum at 277 nm and has no absorbance in the wavelength domain where the peaks of TPPS appear. 

Figure 2 shows that the increase of the concentration of HSA in the samples leads to a bathochromic shift of the peak from 413 nm to 420 nm. A hypochromic effect is observed for the HSA concentration range 0–0.66 µM, but for HSA between 0.66 µM and 2 µM, the absorption peak undergoes a hyperchromic effect. 

These spectral modifications suggest that the two compounds bind, forming a complex. The docking simulation indicates that the complex is formed when TPPS binds to HSA close to Sudlow’s site II.

The strength of an interaction between two compounds, namely a ligand and a receptor, is described by the binding affinity, or Gibbs free energy of binding, and it is linked to the dissociation constant (*K_D_*) [51,52].

In order to determine the binding affinity of TPPS with HSA, we used the Scatchard plot that allows the calculation of the dissociation constant [53]:

(1) n TPPS+HSA ⇔Complex

(2) $$K_{D}=\frac{\left[\mathrm{HSA}\right]\times {\left[\mathrm{TPPS}\right]}^{n}}{\left[Complex\right]}$$

(3) $$\frac{\left[{\mathrm{TPPS}}_{b}\right]}{[{\mathrm{TPPS}}_{f}]\times \left[\mathrm{HSA}\right]}=\frac{n}{K_{D}}-\frac{\left[{\mathrm{TPPS}}_{b}\right]}{\left[\mathrm{HSA}\right]\times K_{D}}$$

where *n* are the number of the binding sites, [TPPS_b_] represents the concentration of TPPS bound molecules, and [TPPS_f_] is the concentration of TPPS free molecules. [TPPS_f_] and [TPPS_b_] are proportional to the absorbances displayed in Figure 2 for the absorption bands at 413 nm and at 420 nm, respectively.

**Figure 3:**
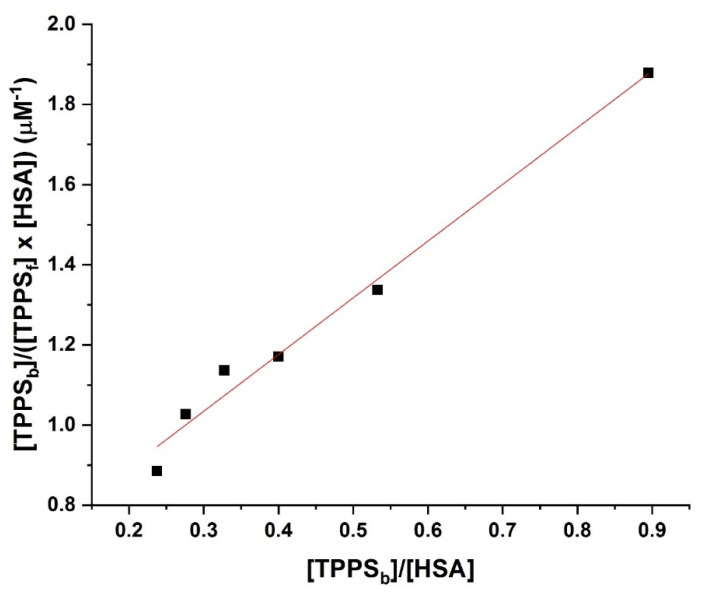
Scatchard plot of TPPS and HSA.

Equation (3) allows us to determine *K_D_* by plotting [TPPS_b_]/([TPPS_f_]×[HSA]) vs. [TPPS_b_]/[HSA] (Figure 3). 

The straight-line fitting of the data in Figure 3 indicate one biding site. If the data had been described by two linear parts on the Scatchard plot, then two binding sites would have been involved [53].

Considering the Equation (3), we determined from Figure 3 *K_D_* as the inverse value of the slope, obtaining a value of 707 nM. 

In this case, *K_D_* is equivalent to the *K_I_*, which is estimated through in silico methods. 

## 4. Discussion

TPPS and temoporfin (the compound with the highest predicted similarity to TPPS according to the Swiss Similarity report) have similar pharmacogenomic profiles. Both compounds interact similarly with the cytochromes CYP1A2, CYP2C19, CYP2C9, CYP2D6, and CYP3A4, which are strongly involved in the metabolism of drugs (Table 3). 

The target predictions indicate that TPPS may interact with four melanoma targets: telomerase reverse transcriptase, C-X-C chemokine receptor type 4, histone deacetylase 1, and galectin-3 (Table 4). The highest probability target is represented by telomerase reverse transcriptase (79%) with a model accuracy of 90%. Telomeres are critical for genetic integrity and decrease with age. The length of telomeres is related to many disorders, including melanoma [54]. Moreover, mutations in the telomerase reverse transcriptase are more common in sun-exposed melanoma than in non-exposed melanoma, and they tend to co-occur with other common melanoma mutations such as BRAF and CDKN2A (Cyclin Dependent Kinase Inhibitor 2A) [55]. According to SuperPred, temoporfin may interact with five melanoma targets: DNA (apurinic or apyrimidinic site) lyase, beta-1 adrenergic receptor, C-X-C chemokine receptor type 4, galectin-3, and toll-like receptor 8. 

ADMET predictions indicate that except for human hepatotoxicity, TPPS is well-tolerated, similar to or better than temoporfin or CPO. TPPS has a low chance of drug-induced liver injury and carcinogenicity, whereas CPO presents a very high (+++) probability for both and temoporfin for drug-induced liver injury (Table 5).

In PDT, protein oxidation occurs most frequently at CYS, MET, TYR, HIS, and TRP AA residues due to their affinity for singlet oxygen. CYS and MET are oxidized to sulfoxides, HIS gives a thermally unstable endoperoxide, TRP converts to N’-formylkynurenine, and phenolic oxidative coupling of TYR is probable [56]. 

Molecular docking studies are often used to predict ligand–target interactions in PDT-related studies [57]. 

**Figure 4:**
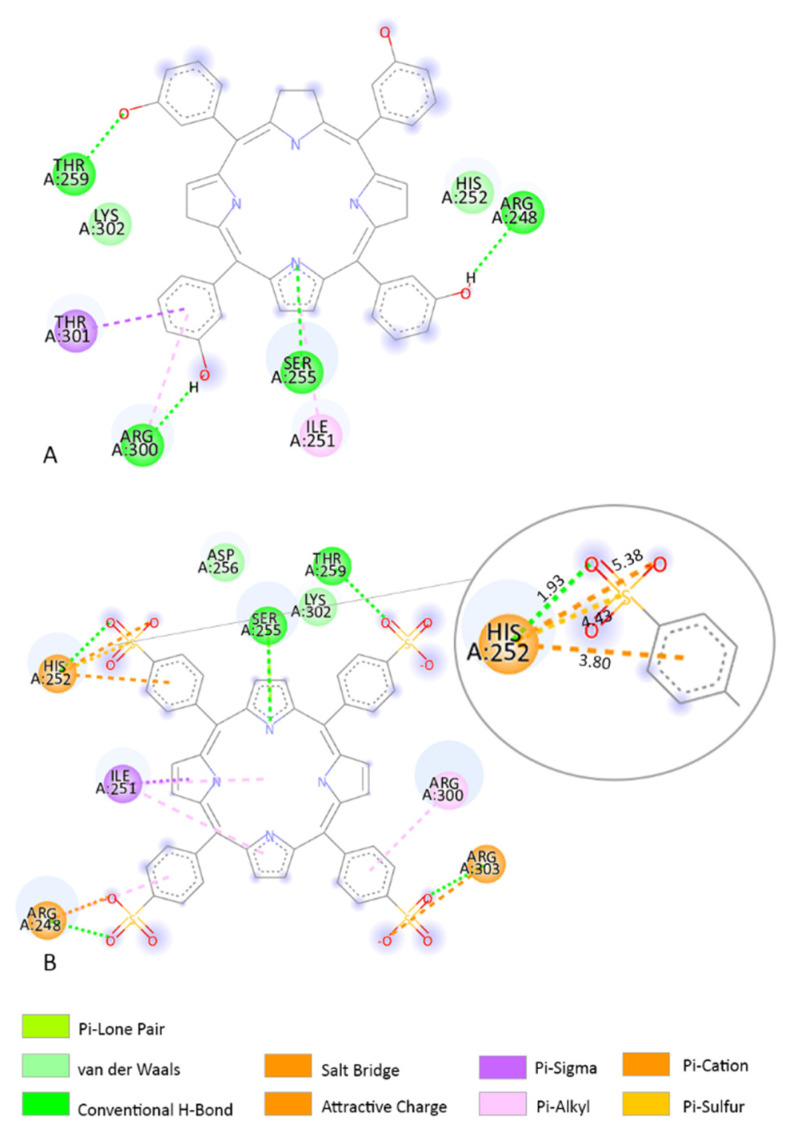
Two-dimensional structures of TPPS, temoporfin, and AA residues from its binding site when interacting with the MCL-1 receptor. (**A**) The interaction between temoporfin and MCl-1; (**B**) the interaction between HIS252 AA residue and TPPS is highlighted in the right corner. HIS252 produces a 1.93 Å-length H-bond interaction with O, a 5.38 Å-length attractive charge with O, a 4.43 Å-length Pi–sulphur interaction, and a 3.80 Å-length Pi–cation interaction. The distance between atoms influences the type of chemical interaction.

Based on our molecular docking findings, we investigated the interaction between the AA residues and TPPS. In addition, we compared the interactions with those of the clinically used compound temoporfin and CPO. MCL-1 overexpression is involved in delaying various stimuli that induce apoptosis and might protect tumour cells from PDT-induced death. MCL-1 in combination with BCL-XL represents a promising target in melanoma. In both in vivo and in vitro tests, downregulation of MCL-1 is correlated with higher efficiency of the photosensitizer in PDT-induced apoptosis [58,59,60,61]. In the MCL-1 receptor, TPPS forms several interactions with HIS252 (Figure 4B). Between HIS252 AA and TPPS are several interactions including a conventional H-bond and an attractive charge with O atoms. HIS252 also forms a Pi-cation interaction and a Pi–sulphur interaction (Figure 4B). At the same receptor, temoporfin binds closely to the TPPS predicted binding sites in the run with the lowest-predicted binding energy (−9.23 kcal/mol). Temoporfin forms H-bond interactions with THR269, SER255, and ARG248 AA residues. Furthermore, temoporfin presents a van der Waals interaction with HIS252 AA residue (Figure 4A).

**Figure 5:**
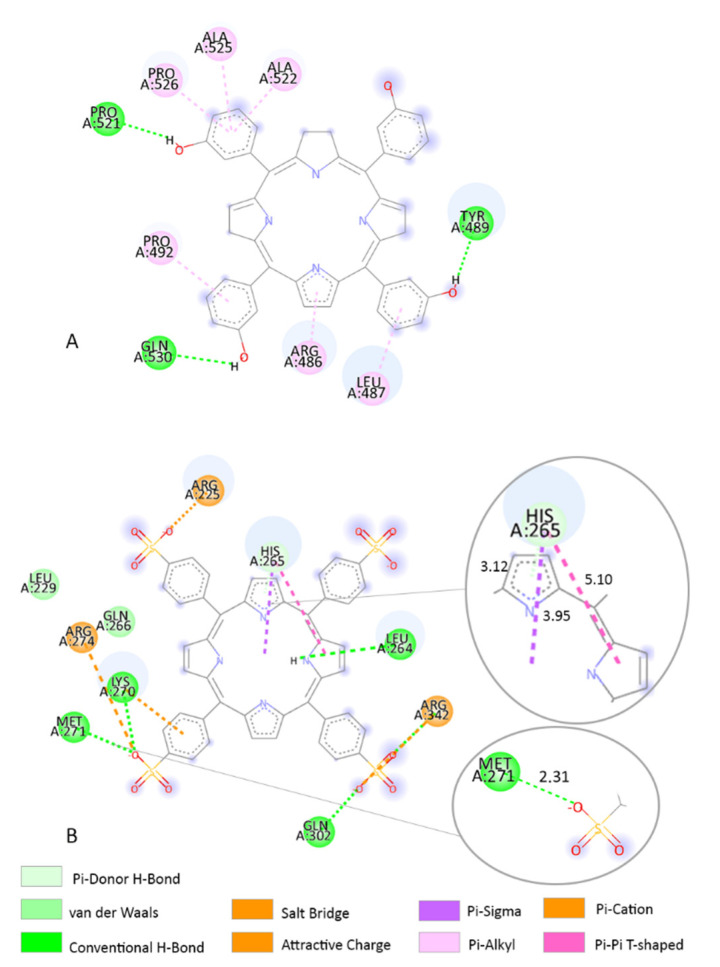
(**A**) Two-dimensional structure of temoporfin and AA residues from its binding site when interacting with the β-catenin receptor. (**B**) Two-dimensional structure of TPPS and AA residues from its binding site. The interaction between HIS265 and MET271 AA residues and TPPS is highlighted in the right corner. HIS265 produces a 3.12 Å-length Pi–Donor H-bond interaction, a 3.95 Å-length Pi–Sigma interaction, and a 5.10 Å-length Pi–Pi T-shaped interaction. MET271 forms a 2.31 Å-length H-bond interaction with the TPPS O atom. The distance between atoms influences the type of chemical interaction.

In the β-catenin receptor, TPPS forms a Pi–Sigma and a Pi–Pi T-shaped interaction with HIS265 AA residue (Figure 5B). In this receptor, MET271 forms a conventional H-bond interaction with the TPPS O atom (Figure 5B). Temoporfin interacts with the β-catenin receptor in a different binding site and forms an H-bond interaction with AA residue TYR489 (Figure 5A). 

**Figure 6:**
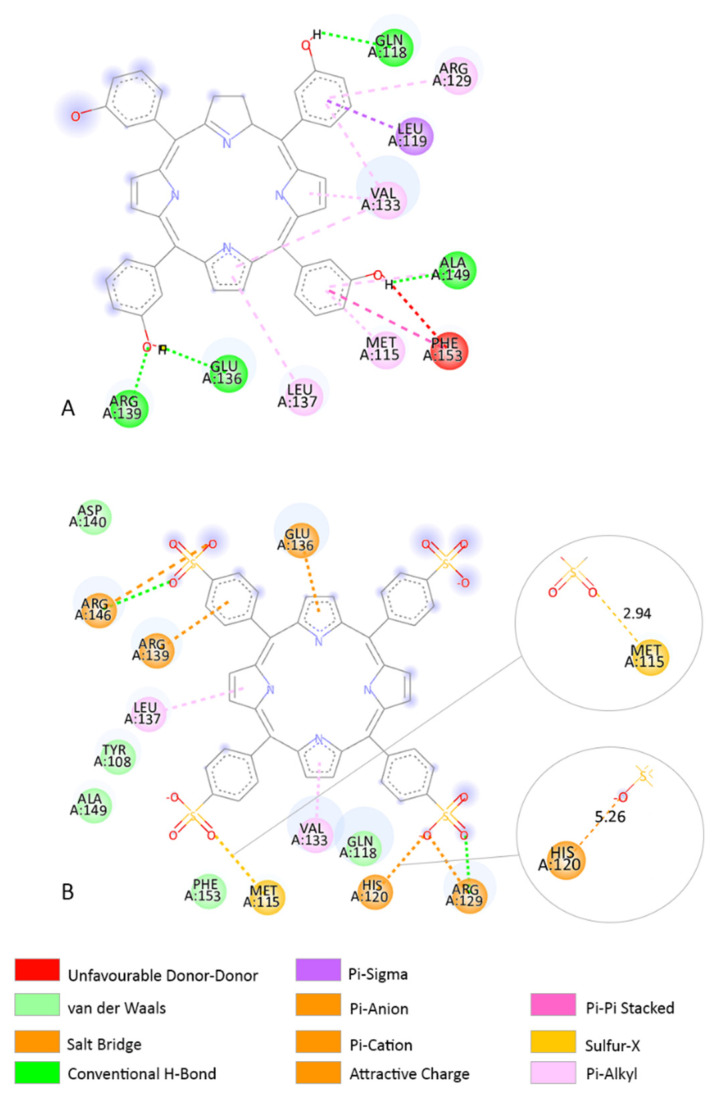
(**A**) Two-dimensional structure of TPPS and AA residues from its binding site when interacting with the BCL-2 receptor. (**B**) Two-dimensional structure of TPPS and AA residues from its binding site when interacting with the BCL-2 receptor. The interaction between HIS120 and MET115 AA residues and TPPS is highlighted in the right corner. HIS120 produces a 5.26 Å-length attractive charge with O atom. MET115 form a 2.94 Å-length sulphur–X interaction with TPPS O atom. The distance between atoms influences the type of chemical interaction.

In the BCL-2 receptor, TPPS forms a sulphur–X interaction with the MET115 AA residue (Figure 6B). Methionine is an AA that contains sulphur and is easily oxidized [62]. Moreover, in this receptor, HIS120 forms an attractive charge interaction with the O atom (Figure 6B). Temoporfin interacts with the BCL-2 receptor close to the TPPS binding site and forms similar interactions with LEU137 and VAL133 AA residues (Figure 6A). Temoporfin forms a Pi–alkyl interaction with MET115 AA residue.

Our molecular docking studies indicate that TPPS has the lowest binding affinity when interacting with BCL-2. Xue et al. study showed that BCL-2 protein is destroyed by a phthalocyanine photosensitizer during PDT [63]. Moreover, the involvement of BCL-2 and Bax in PDT-mediated cell death was underlined by Srivastava et al. [64] and Kim et al. [65].

**Figure 7:**
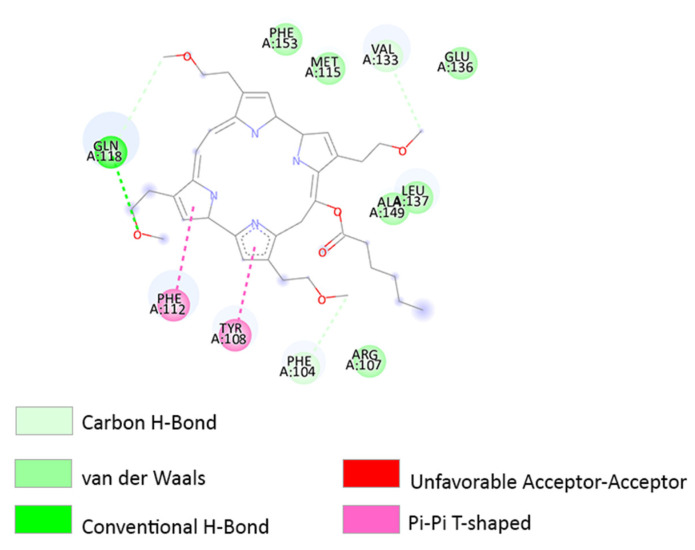
Two-dimensional structure of CPO and AA residues from its binding site when interacting with the BCL-2 receptor.

Donohoe et al. [66]’s study indicated that the degradation of anti-apoptotic proteins, including BCL-2, is linked to the accumulation of photosensitizers in mitochondria. According to the study membrane degradation can be caused by CPO and temoporfin PS [66]. When interacting with the same receptor, CPO is also docked close to TPPS predicted sites (Figure 7). TPPS and CPO have interactions with eight AA: TYR108, MET115, GLN118, VAL133, GLU136, LEU137, ALA149, and PHE153 (Figure 6B and Figure 7). Moreover, all the porphyrin derivatives form interactions with MET115 AA residue: CPO forms a van der Waals interaction, TPPS a sulphur–X interaction, and temoporfin a Pi–alkyl interaction (Figure 6B and Figure 7).

Since HSA is the primary drug carrier, the interaction between HSA and TPPS was studied both in silico and experimental by UV–vis absorption spectroscopy. The in silico evaluation shows that at a temperature of 25 degrees Celsius, the lowest binding affinity is −8.35 kcal/mol (Table 6), and the estimated *K_I_* is 760 nM. This is supported by the experimental constant (707 nM) determined by Scatchard plot of the spectroscopic data. 

**Figure 8:**
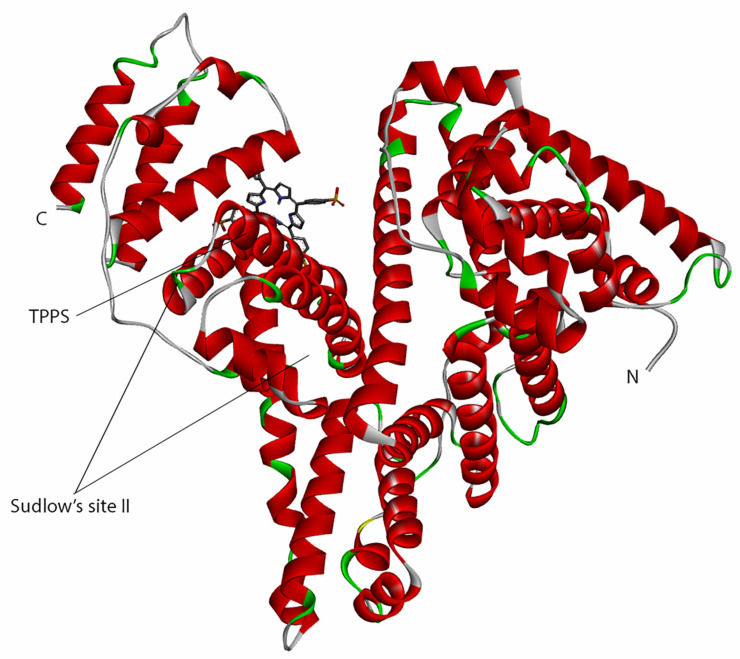
HSA 3D structure in interaction with TPPS.

The interactions of the AA residues from the predicted binding site show that in the run with the lowest-predicted binding energy, the TPPS compound is bound close to Sudlow’s site II (Figure 8).

The interactions between other photosensitizers and HSA have been computed or experimentally determined in several studies [67,68,69]. In comparison with our results, Guevara et al. study showed that three glycosylated photosensitizers bind to HSA close to Sudlow’s site I and form strong interactions with TRP214 [67]. Zheng et al. predicted the interaction of tetra-(p-sulfoazophenyl-4-aminosulfonyl)-substituted aluminium(III) phthalocyanine photosensitizer with HSA. Their study also showed that the photosensitizer interacts with TRP214 AA residue [68]. Szafraniec’s study showed that two derivatives of chlorophyll bind to HAS to Sudlow’s sites I and II [69]. Escobar et al. predicted and determined a high interaction of tetracarboxyphenyl porphyrin with bovine serum albumin also near the Sudlow’s site II, similar to our study [70].

## 5. Conclusions

When compared to temoporfin and CPO, TPPS shows that it is a well-tolerated molecule with low or equivalent toxicity and a similar pharmacogenomic profile. TPPS has low estimated binding energies when interacting with our selected protein targets. The lowest-predicted binding energies of TPPS are obtained when interacting with A1 and BCL-B receptors. When interacting with BCL-2, MCL-1, and β-catenin, TPPS forms interactions with MET and HIS AA residues. TPPS showed a binding affinity that indicates biological activity on HSA. The similarity between experimental and computed *K_I_* indicate that TPPS may bind to HSA near Sudlow’s site II, as predicted through molecular docking. 

## Figures and Tables

**Table 2:** The small compounds used in this article, the SMILES code, and their 2D structure.

Compound Name and SMILES Code	2D Structure
TPPS: O=S(=O)([O-])c9ccc(c7c1ccc(n1)c(c2ccc(S(=O)(=O)[O-])cc2)c3ccc([nH]3)c(c4ccc(S(=O)(=O)[O-])cc4)c5ccc(n5)c(c6ccc(S(=O)(=O)[O-])cc6)c8ccc7[nH]8)cc9	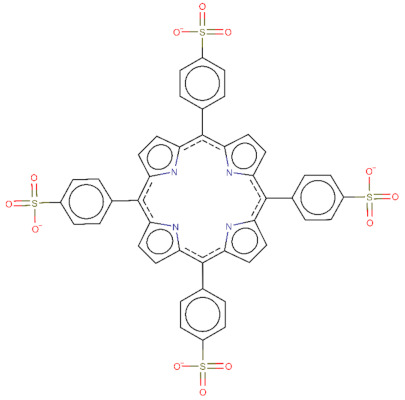
Temoporfin: C1CC2=NC1=C(C3=CC=C(N3)C(=C4C=CC(=N4)C(=C5C=CC(=C2C6=CC(=CC=C6)O)N5)C7=CC(=CC=C7)O)C8=CC(=CC=C8)O)C9=CC(=CC=C9)O	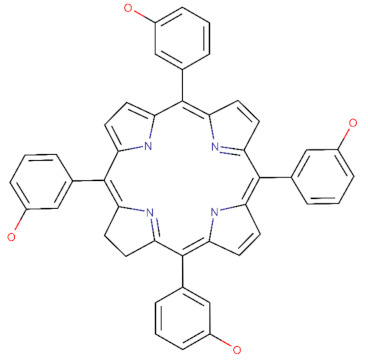
CPO: O(C(=O)CCCCC)\C\1=C/2\NC(C=C\2CCOC)C2N=C(\C=C/c3[nH]c(cc3CCOC)C3=N\C(=C/1)\C(=C3)CCOC)C(=C2)CCOC	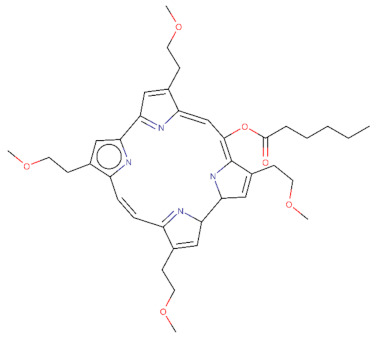

**Table 3:** TPPS, temoporfin, and CPO predictions (pred) and probabilities (prob) to be inactive or active compounds on CYPs targets using MACCS and Morgan fingerprints [28].

Target	Fingerprint	TPPS		Temoporfin		CPO	
		Pred	Prob	Pred	Prob	Pred	Prob
CYP1A2	MACCS	Inactive	0.799	inactive	0.599	Inactive	0.771
CYP1A2	Morgan	Inactive	0.563	inactive	0.531	Inactive	0.768
CYP2C19	MACCS	Inactive	0.698	inactive	0.547	Inactive	0.779
CYP2C19	Morgan	Inactive	0.829	inactive	0.847	Inactive	0.729
CYP2C9	MACCS	Active	0.628	active	0.571	Inactive	0.607
CYP2C9	Morgan	Inactive	0.6	inactive	0.673	Inactive	0.782
CYP2D6	MACCS	Inactive	0.762	inactive	0.612	Inactive	0.59
CYP2D6	Morgan	Inactive	0.802	inactive	0.509	Inactive	0.548
CYP3A4	MACCS	Inactive	0.737	inactive	0.598	Inactive	0.698
CYP3A4	Morgan	Inactive	0.711	inactive	0.623	Inactive	0.504

**Table 4:** Temoporfin and TPPS predicted targets and their therapeutic indications [29].

Temoporfin			
Target Name	Therapeutic Indication	Prob	Accuracy
DNA (apurinic or apyrimidinic site) lyase	Glioma, melanoma, ocular cancer, solid tumour/cancer	96.37%	91.11%
Beta-1 adrenergic receptor	Melanoma	83.6%	95.56%
C-X-C chemokine receptor type 4	Acute lymphoblastic leukaemia, acute myeloid leukaemia, B-cell chronic lymphocytic leukaemia, breast cancer, haematological malignancy, melanoma, Merkel cell carcinoma, multiple myeloma, myelodysplastic syndrome, non-Hodgkin’s lymphoma pancreatic cancer, renal cell carcinoma, sarcoma solid tumour/cancer	76.64%	93.1%
Galectin-3	Melanoma	73.88%	96.9%
Toll-like receptor 8	Melanoma, solid tumour/cancer	55.13%	96.25%
**TPPS**			
**Target Name**	**Therapeutic indication**	**Prob**	**Accuracy**
Telomerase reverse transcriptase	Acute myeloid leukaemia, brain cancer, breast cancer, head and neck cancer, liver cancer, melanoma, multiple myeloma, non-small-cell lung cancer, ovarian cancer, pancreatic cancer, prostate cancer, solid tumour/cancer	79%	90%
C-X-C chemokine receptor type 4	Acute lymphoblastic leukaemia, acute myeloid leukaemia, B-cell chronic lymphocytic leukaemia, breast cancer, haematological malignancy, melanoma, Merkel cell carcinoma, multiple myeloma, myelodysplastic syndrome, non-Hodgkin’s lymphoma pancreatic cancer, renal cell carcinoma, sarcoma solid tumour/cancer	74%	93.1%
Histone deacetylase 1	Acute myeloid leukaemia, breast cancer, colorectal cancer, cutaneous T-cell lymphoma, diffuse large B-cell lymphoma, hepatocellular carcinoma, leukaemia, melanoma, Merkel cell carcinoma, multiple myeloma, non-small-cell lung cancer, ovarian cancer, peripheral T-cell lymphoma, renal cell carcinoma, solid tumour/cancer	60%	96%
Galectin-3	Melanoma	55%	96.9%

**Table 5:** The ADMET prediction for TPPS, temoporfin, and CPO. The prediction probability is represented as a score between 0 and 1, where 0 is the lowest chance (“−” represent a low probability and “+” a high probability) [27].

Prediction Probability	TPPS	Temoporfin	CPO
hERG blockers	0.3–0.5 (−)	0.3–0.5 (−)	0.1–0.3 (−−)
Human hepatotoxicity	0.9–1.0 (+++)	0.1–0.3 (−−)	0.3–0.5 (−)
Drug-induced liver injury	0.1–0.3 (−−)	0.9–1.0 (+++)	0.9–1.0 (+++)
Carcinogenicity	0.3–0.5 (−)	0–0.1 (−−−)	0.9–1.0 (+++)
Respiratory toxicity	0.9–1.0 (+++)	0.9–1.0 (+++)	0.9–1.0 (+++)
Androgen receptor ligand-binding domain	0–0.1 (−−−)	0.1–0.3 (−−)	0–0.1 (−−−)
Aryl hydrocarbon receptor	0.3–0.5 (−)	0.7–0.9 (++)	0.9–1.0 (+++)
Oestrogen receptor ligand-binding domain	0.7–0.9 (++)	0.9–1.0 (+++)	0.7–0.9 (++)
Peroxisome proliferator-activated receptor gamma	0–0.1 (−−−)	0.3–0.5 (−)	0–0.1 (−−−)
Heat-shock factor response element	0–0.1 (−−−)	0.7–0.9 (++)	0–0.1 (−−−)
Mitochondrial membrane potential	0.7–0.9 (++)	0.9–1.0 (+++)	0.5–0.7 (+)
P53	0.5–0.7 (+)	0.9–1.0 (+++)	0.7–0.9 (+++)
Acute-toxicity rule	0 alert	0 alert	0 alert

**Table 6:** Biological target, the lowest EFEB, and *K_I_* for TPPS and the clinically used compound, temoporfin, predicted using Autodock software.

Target	TPPS EFEB (kcal/mol)	TPPS *K_I_* (nM)	Temoporfin EFEB (kcal/mol)	Temoporfin *K_I_* (nM)
BCL-2	−7.90	1610	−10.26	30.08
BCL-B	−11.49	3.76	−10.83	11.55
BCL-xL	−8.81	349.53	−9.19	184.88
MCl-1	−9.95	51.27	−9.23	171.65
A1	−14.99	0.01023	−10.63	16.05
BCL-W	−9.05	233.04	−9.92	53.25
β-catenin	−9.81	64.39	−7.35	4070
NFKB	−10.92	9.78	−9.84	61.66
Fas	−7.69	2290	−10.20	33.26
EIF2AK1	−10.99	8.77	−11.44	4.13
HSA	−8.35	761.92	-	

## Data Availability

Not applicable.
